# PREDICTORS OF 400M WALKING TIME: THE STUDY OF MUSCLE MOBILITY AND AGING (SOMMA)

**DOI:** 10.1093/geroni/igac059.3009

**Published:** 2022-12-20

**Authors:** Steve Cummings, Sheena Patel, Peggy Cawthon, Paul Coen, Russell T Hepple, Theresa Mau, Anne Newman

**Affiliations:** San Francisco Coordinating Center, San Francisco, California, United States; CPMC Research Institute, San Francisco, California, United States; University of California, San Francisco, San Francisco, California, United States; AdventHealth, Orlando, Florida, United States; University of Florida, Gainesville, Florida, United States; San Francisco Coordinating Center, San Francisco, California, United States; University of Pittsburgh, Pittsburgh, Pennsylvania, United States

## Abstract

Longer 400m walking time has been strongly associated with overall mortality and many other aging-related outcomes in prior studies. Inability to walk 400m defines ‘mobility disability’. We assessed characteristics that might contribute to 400m walking time in SOMMA, a cohort of 879 men and women over age 70. We excluded people with a gait speed < 0.6 m/s. The analysis included 49 factors representing several domains: muscle mass and strength, body composition, oxygen delivery, muscle mitochondrial ATP generation, fitness by cardiopulmonary exercise testing (VO2 peak), fatigability by questionnaire, usual physical activity, depressed mood, cognitive performance, lower extremity pain, lower extremity sensation, smoking, wealth and income, and general health. We used both elastic net and forward step-wise regression to generate multivariate models. Factors associated with longer (worse) walking time in both elastic net and forward step-wise regression models were: *weaker leg power, *fatigue during treadmill testing, *higher BMI, *greater self-reported fatigability, *worse performance on the timed DSST test, *fewer hours spent walking, *taking more medications, *older age, *poorer fitness (VO2peak), *more joint stiffness, *smoking, and *reduced peripheral sensation. These results indicate that 400m walk time is influenced by numerous factors. Some are also associated with overall mortality and aging outcomes and therefore they might underlie the prognostic value of the 400m walk. Additionally, some of the predictors could be improved, such as leg strength, cardiopulmonary fitness, more time walking, quitting smoking, and treatment of joint stiffness, perhaps reducing the risk of mobility disability.

